# Giant right coronary artery aneurysm presenting with non-ST elevation myocardial infarction and severe mitral regurgitation: a case report

**DOI:** 10.1186/1752-1947-5-442

**Published:** 2011-09-07

**Authors:** Justin Nazareth, Laurence Weinberg, Jon Fernandes, Phil Peyton, Sivan Seevanayagam

**Affiliations:** 1 Department of Anesthesia, Austin Hospital, 145 Studley Road, Heidelberg, Victoria, 3084, Australia; 2 Department of Cardiac Surgery, Austin Hospital, 145 Studley Road, Heidelberg, Victoria, 3084 Australia

## Abstract

**Introduction:**

Coronary artery aneurysms are seen in 1.5-5% of patients presenting for coronary angiography, but giant aneurysms, defined as being greater than 2 cm in diameter, are rare. Given the paucity of cases and limited experience in diagnosis and management of the disease, each case is a learning tool in itself.

**Case presentation:**

We report the rare case of a 78-year-old Caucasian man who presented to a peripheral emergency department with chest pain and was subsequently found to have a giant right coronary artery aneurysm. Following initial investigation and treatment he was referred to our hospital for definitive management.

**Conclusion:**

The case described illustrates one of the varied presentations and subsequent management of an ill-defined and heterogeneous disease process. Given the limited experience with giant aneurysms in the coronary circulation, this case provides valuable insight into the clinical presentation of the disease and gives an example of the management of the most recent such case at our hospital.

## Introduction

Coronary artery aneurysms are a relatively common entity. However, giant aneurysms (> 2 cm diameter) are rare [1,2]. Most are atherosclerotic in nature, but the exact mechanism leading to the development of ectasia in these vessels is unknown. Evidence suggests that contributory factors include a combination of genetic predisposition, common risk factors for coronary artery disease, direct arterial wall damage and abnormal vessel wall metabolism [1-4]. The low-recorded incidence of these giant aneurysms means the clinical presentation and management of this pathology is poorly defined.

We present the case of a 78-year-old man with a previously undiagnosed giant right coronary artery aneurysm who presented to a peripheral emergency department with chest pain. He was subsequently referred to our hospital for definitive management.

## Case presentation

A 78-year-old Caucasian man with a history of myocardial infarction (MI) presented to a peripheral hospital with atypical chest pain and sudden onset of dyspnea one week after an elective inguinal hernia repair. Clinical examination revealed dual heart sounds with no murmurs and reduced air-entry bilaterally. Initial investigations revealed a modest elevation in cardiac enzymes (troponin I 0.8 ng/mL, normal value < 0.01 ng/mL). An electrocardiogram at admission showed sinus rhythm with no evidence of right heart strain or new ischemic changes. His initial chest radiograph showed no clear cause for his symptoms. A computed tomography pulmonary angiography (CTPA) excluded a postoperative pulmonary embolism. He was treated empirically for an acute coronary syndrome with a loading dose of aspirin (150 mg) and therapeutic anticoagulation with daily subcutaneous enoxaparin (80 mg).

**Figure 1:**
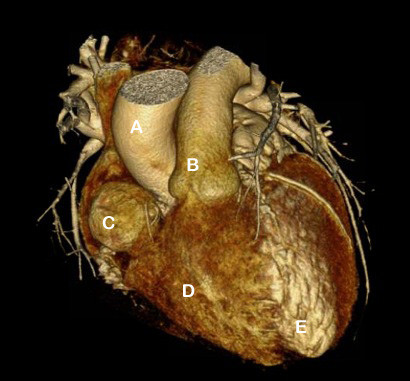
**Cardiac CT image exhibiting the giant right coronary artery aneurysm**. A- aorta; B - pulmonary artery; C - right coronary artery aneurysm; D - right ventricle; E - left ventricle.

**Figure 2:**
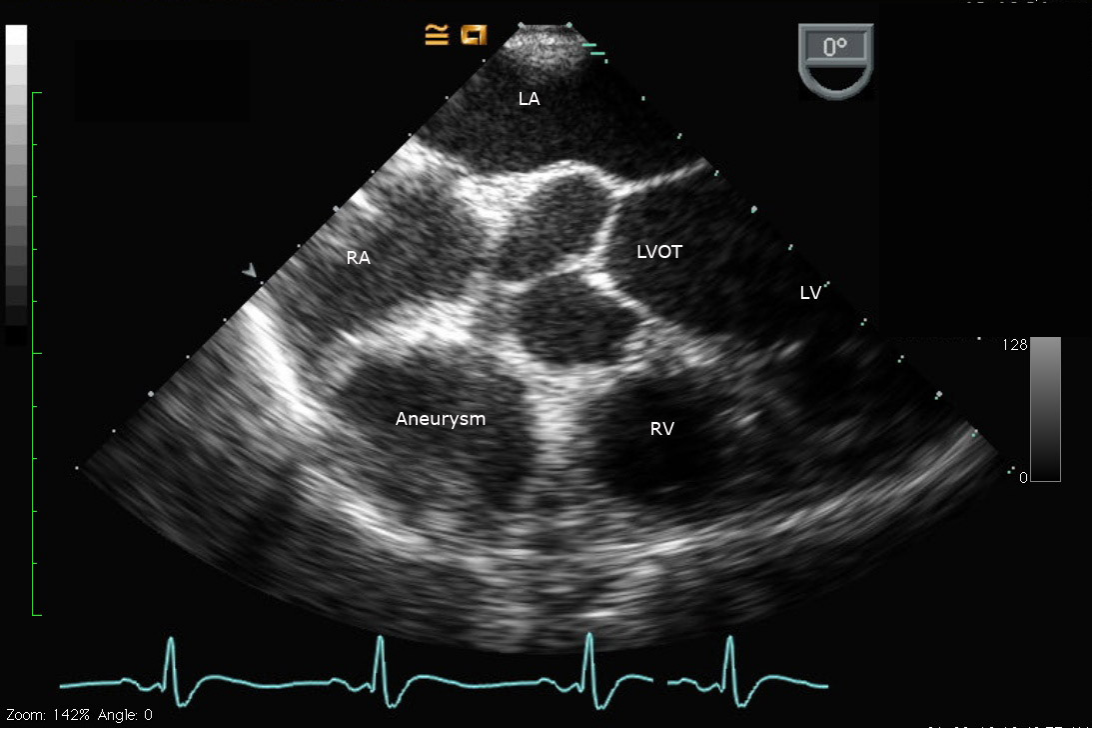
**Midesophageal transesophageal modified short axis view of the right ventricular outflow tract**. RA - right atrium; RV - right ventricle; LV - left ventricle; LVOT - left ventricular outflow tract.

**Figure 3:**
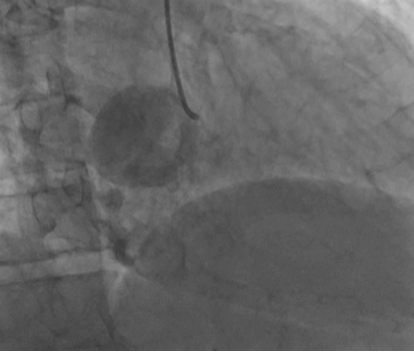
**Right coronary artery angiogram**. The giant aneurysm is seen at the catheter tip.

Although CTPA excluded a pulmonary embolism, it did identify an aneurysm, 4 cm in diameter, extending from the dominant right coronary artery. The artery itself appeared to remain patent. The aneurysm was further demarcated with cardiac computed tomography (CT) (Figure 1), transesophageal echocardiography (Figure 2) and coronary angiography (Figure 3).

The echocardiogram revealed a 4 cm aneurysm of his right coronary artery, a dilated left ventricle with severe segmental dysfunction, biatrial enlargement and severe mitral regurgitation (Figure 2). The mechanism of the mitral regurgitation was secondary to ischemia from the acute MI. Although the mitral valve leaflets and subvalvular apparatus appeared relatively normal there were also minor degenerative changes of the mitral valve itself. The coronary angiogram (Figure 3) illustrated the aneurysm, whose lumen appeared clear with no evidence of thrombus, in addition to left main disease. It was decided that our patient would undergo coronary artery bypass surgery, with exclusion of the aneurysm and mitral valve annuloplasty.

**Figure 4:**
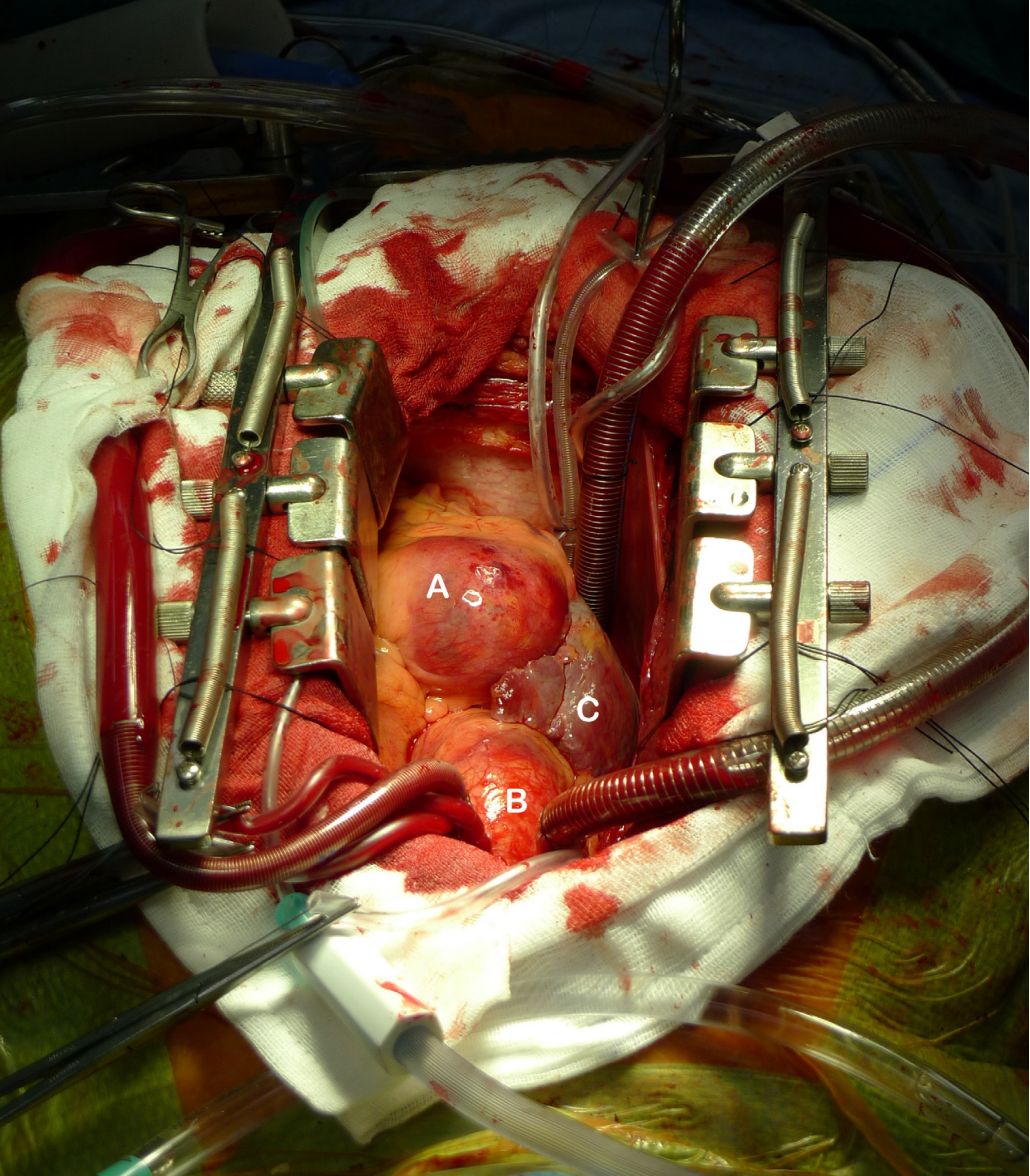
**Intraoperative images displaying the giant right coronary artery aneurysm**. A - right coronary artery aneurysm; B - aorta, C - right atrium.

Median sternotomy was performed with subsequent pericardiotomy. The conduits (left internal mammary artery, left radial artery and right radial artery) were then harvested. After cardiopulmonary bypass was established, and cardioplegia administered, the conduits were grafted in place with the following configuration: left internal mammary artery to the first diagonal branch of his left anterior descending artery; left radial artery to his posterior descending artery; right radial artery to his left anterior descending artery. His left atrium was then opened to expose the mitral valve. Mitral valve annuloplasty was performed using a saddle ring. After testing to show no further mitral regurgitation, his left atrium was closed. Finally the right coronary artery aneurysm (Figure 4) was opened and excluded with closure of the coronary ostium within. After mitral valve repair and revascularization, transesophageal echocardiographic assessment determined that the mitral valve was competent with no insufficiency or stenosis. There was no systolic anterior motion or left ventricular outlet tract obstruction from the anterior mitral valve leaflet, and no new regional wall motion abnormalities in the lateral wall or inferoposterior regions to suggest a circumflex artery injury following repair. Following re-establishment and stabilization of our patient's normal circulation and closure of his chest, he was transferred to our intensive care unit. He was discharged to rehabilitation nine days later.

## Discussion

Coronary artery aneurysm is defined as a localized area of dilatation exceeding the diameter of the adjacent normal arterial segment by 50%. Ectasia of the coronary vessels is relatively common, being seen in 1.5-5% of patients who present for angiography [1-4]. Giant aneurysms, defined as those aneurysms greater than 2 cm in diameter, are rare [1,2]. Giant coronary artery aneurysms have been identified throughout the coronary arterial circulation, with the right coronary artery being more commonly affected than the left [4,5]. On several occasions, multiple giant aneurysmal arteries have been identified in a single patient [1,4-6].

The precise mechanism leading to the development of ectasia in the coronary circulation is unknown. Genetic predisposition, common risk factors for coronary arterial disease, direct vessel injury and abnormal vessel wall metabolism are thought to play a part [1-3]. Atherosclerosis, either by stenosis with poststenotic dilatation or by direct destruction of the arterial lumen, accounts for 50% of coronary artery aneurysms. The other well-documented association is with Kawasaki disease. The remainder are thought to be associated with other methods of primary luminal injury (dissection, coronary angioplasty with or without stent insertion, other forms of vasculitis or mycotic emboli) or to be congenital [1-4]. Some authors suggest that giant aneurysms are more likely to be congenital rather than due to atherosclerosis, unlike their smaller counterparts [2]. In the case of our patient, given the history of previous MI and significant atherosclerosis seen on angiography, the likelihood could be that the aneurysm was a complication of an atherosclerotic process.

The literature available gives the impression of a pathological entity that is highly variable in its symptomatology and morbidity and thus its diagnosis and subsequent management. Chest pain, angina, MI, heart failure, the presence of a mediastinal mass, superior vena cava obstruction and even hemoptysis have been described as presenting problems, however no reliable clinical features to identify a coronary artery aneurysm have been described, and in fact most patients are asymptomatic [1-3,5-8]. The presence of a diastolic or continuous murmur is occasionally noted, and large or calcified aneurysms may be visualized on chest radiography or echocardiogram, but coronary angiography is required for definitive diagnosis and characterization of the aneurysm [1,5].

Untreated or undiagnosed aneurysms can be complicated by rupture, thromboembolic phenomenon and, more rarely, fistulization into one of the cardiac chambers [1,2,5]. These cases may present *in extremis *with tamponade, heart failure or even sudden death [5]. Prognosis is controversial, however overall five-year survival is in the region of 71% [9].

Management may be medical or surgical with the same outcomes in mind--reduction or elimination of spontaneous rupture risk and restoration of distal flow [7,10]. Medical management with anticoagulation or antiplatelet agents has a role in these patients, as does the insertion of a covered stent to exclude aneurysmal flow, however the literature suggests that surgical intervention is the preferred treatment pathway for giant coronary artery aneurysms [1,5,10]. Surgical management requires median sternotomy, cardiopulmonary bypass and coronary artery bypass grafting in conjunction with exclusion of the aneurysm. A femoral approach may be made to cardiopulmonary bypass to first decompress the aneurysm and ventricle before opening the chest, an approach which is felt by some authors to be more prudent when operating on very large aneurysms [2]. In addition, consideration should always be given to opening giant aneurysms immediately after cardioplegic arrest, prior to performing any coronary artery bypass grafting, as the majority of large aneurysms may contain a large amount of layered thrombus within, and any manipulation of the heart, even in the arrested state, poses a danger of the embolus flowing to the distal segment of the coronary artery.

## Conclusion

Giant coronary artery aneurysms are a rare clinicopathological entity and thus there is a dearth of information regarding their presentation, diagnosis and subsequent management. Given this, each case deserves attention so as to build on the database of information accessible by those who may encounter a similar case in the future. Although our case described above is not unique, it does represent an uncommon presentation of a rare disease process. We put forward this case and the associated literature review as an example of how a giant coronary artery aneurysm was managed at our hospital in the hope that it may aid clinicians in the future.

## Consent

Written informed consent was obtained from the patient for publication of this case report and any accompanying images. A copy of the written consent is available for review by the Editor-in-Chief of this journal.

## Competing interests

The authors declare that they have no competing interests.

## Authors' contributions

Together JN and LW collated the information regarding the case. LW and PP were the anesthetists involved in the case. SS was the cardiac surgeon. JN completed the literature review and wrote the manuscript. LW, PP, JF and SS contributed to writing the manuscript. All authors read and approved the final manuscript.
